# Thoracic Spinal Anesthesia for Laparoscopic Cholecystectomy: An Observational Feasibility Study

**DOI:** 10.7759/cureus.36617

**Published:** 2023-03-24

**Authors:** Richa Chandra, Gaurav Misra, Gopal Datta

**Affiliations:** 1 Anaesthesiology, Subharti Medical College , Meerut, Bareilly, IND; 2 Anesthesia, Shri Ram Murti Smarak Institute of Medical Sciences (SRMS IMS), Bareilly, IND; 3 Laparoscopic Surgery, GD Hospital, Bareilly, IND

**Keywords:** neuraxial anaesthesia, laparoscopy, segmental block, laparoscopic cholecystectomy, thoracic spinal anaesthesia

## Abstract

Background: The expanding horizons of the application of Segmental Thoracic Spinal anesthesia in day-to-day anesthesia practice prompted us to perform this study in a large subset of healthy patients with the aim of determining the feasibility, safety, advantages, and complications of this mode of anesthesia.

Material and methods: The prospective observational study was conducted from April 2020 to March 2022, 2.146 patients with symptoms of cholelithiasis and planned for laparoscopic cholecystectomy were included in this study, and 44 patients from this study were excluded due to pre-defined exclusion criteria. The patients belonging to ASA lIl, lV, severe cardiovascular or renal disability, on beta blockers, coagulation anomaly, spinal deformities, or previous spine surgeries were not included in the study. The patients exhibiting allergy to local anesthetics, requiring more than two attempts for the procedure, patchy or inadequate effects after spinal anesthesia, or change in the plan of surgery intraoperatively were also excluded from the study. All other patients were given subarachnoid block at T10-T11 intervertebral space with 26G Quincke needle and Inj. Bupivacaine Heavy (0.5%) 2.4 mL with 5µg of Dexmedetomidine. Intraoperative parameters, number of attempts, the incidence of paresthesia during the procedure intraoperative and postoperative complications, and patient satisfaction were evaluated and recorded.

Results: Spinal anesthesia was successful in 2,074 patients and was achieved in a single attempt of procedure in 92% of patients. The incidence of paresthesia during needle insertion was 5.8%. Hypotension was observed in 18% of patients, bradycardia (13%), and nausea (10%) in a few patients, with shoulder tip pain in only 6% of patients. The majority of patients (94%) were “very satisfied” with the procedure. There were no episodes of any adverse event during the postoperative period.

Conclusion: Thoracic spinal anesthesia is a regional anesthesia technique practically feasible for healthy patients undergoing laparoscopic cholecystectomy with a manageable incidence of intraoperative complications and no evidence of any neurological complications. It has the advantage of providing manageable hemodynamics, minimal postoperative complication, and an acceptable degree of patient satisfaction.

## Introduction

The introduction of the laparoscopic approach to various surgeries has changed the era in the medical field owing to its advantage of less tiny scar size, significantly reduced bleeding, minimal perioperative complications, and short hospital stay.

Spinal anesthesia was introduced by Bier in 1898, traditionally this term has been restricted to lumbar spinal anesthesia (LSA). LSA has been extensively studied and utilized for laparoscopic cholecystectomies [1]. Since the introduction of thoracic spinal anesthesia (TSA), it had been an area of research interest, although surrounded by controversies. It has been the most controversial technique in recent times nevertheless it can open new horizons in the practice of anesthesia in current times. Jonnesco was the pioneer who introduced the thoracic approach for regional anesthesia and advocated the use of two approaches for “general spinal anesthesia.” “General spinal anesthesia” term was used for the subarachnoid puncture in the thoracic region [2]. The first approach was for surgeries involving skull, head, neck, and upper limb surgeries and the puncture site was between the first and second dorsal vertebrae. For surgeries involving the whole lower part of the body, the puncture site was defined between the last dorsal and first lumbar vertebrae [2].

Performing regional spinal anesthesia above the termination of the conus medullaris has a theoretically defined risk of injuring the spinal cord. Subarachnoid myelography by neurologists in past was performed by utilizing cervical and thoracic punctures of subarachnoid spaces [3]. However, the thoracic approach has been proven as an effective and feasible method for various thoracic, abdominal, and lower limb surgeries with an acceptable safety profile [4]. Mahmoud et al. used spinal anesthesia at T10 thoracic level for successfully conducting breast surgeries; however the incidence of hemodynamic instability was observed to be 16% [5]. Based on the appreciable effects of TSA, the present study was planned to evaluate its effectiveness of it in patients planned for laparoscopic cholecystectomy. The primary outcome of the study was the number of attempts taken for the procedure together with the patient satisfaction and the secondary outcome was the adverse effects if any occurred during/after the procedure.

## Materials and methods

The prospective observational study was carried out at tertiary care NABH accredited Hospital with approval by the Research and Ethical Committee (vide letter no. GDS/2020/011) and after obtaining written informed consent from the enrolled patients. Considering the higher incidences of hemodynamic instability in the existing literature, consent from the patients regarding the same was also obtained in the study. The study included 2146 patients aged 40-66 years with American Society of Anesthesiologists (ASA) physical status l, ll scheduled for Elective Laparoscopic Cholecystectomy from April 2020 to March 2022. Patients belonging to ASA lIl, lV, severe cardiovascular or renal disability, on beta blockers, coagulation anomaly, spinal deformities, or previous spine surgeries were not included in the study. The patients exhibiting allergy to local anesthetics requiring more than two attempts for the procedure, patchy or inadequate effects after spinal anesthesia, or change in the plan of surgery intraoperatively were also excluded from the study.

In the preoperative visit, the procedure and intended anesthetic technique were explained to all the patients. All the patients underwent standard preoperative evaluation, including history, physical and clinical examination, and laboratory investigations including ECG and chest x-ray were advised and checked. All patients were admitted a day prior to surgery. The premedications advised to patients a night before surgery was Tab. Alprazolam 0.5 mg and Tab. Pantoprazole 40 mg at night before surgery and was kept nil per oral for 8 hrs.

On arrival to the pre-operative room, an 18-G intravenous cannula was preferably secured on the left dorsal side of the hand. A volume preload of 10 ml/kg lactated Ringer's solution was administered to patients before the commencement of the procedure. In the operating room, standard monitors were attached (ECG, noninvasive blood pressure, pulse oximeter and end tidal CO_2_). The patient was placed in the sitting position with the head and spine flexed on a pillow to perform the block.

Under aseptic precautions, in a sitting position, a subarachnoid block was given at T10-T11 or T11-T12 intervertebral space by using 26 gauze Quincke Babcock needle. After piercing the ligamentum flavum, we removed the stylet and further advances the needle, and look for free flow of the CSF. All the patients received 2.4 mL hyperbaric Bupivacaine (0.5%) with 5µg of Dexmedetomidine. The height of the block was adjusted by controlling the table remote at 10^0^-20^0^ head tilt. Supplemental oxygen was administered (5-6 L/min) with the aid of an oxygen face mask. The number of needle insertion attempts required and the occurrence of paresthesia during either needle insertion or drug injection were both recorded. The onset of action and level of sensory block (both upper and lower level) was assessed by the pinprick method 5 min after performing the block and reassessed every 5 min for 15 min until the establishment of the desired level. The maximum upper and lower sensory levels reached after 15 min were recorded (target block level was from T4- L2).

Vital signs such as heart rate, respiratory rate, and oxygen saturation were continuously monitored. Systolic blood pressure (SBP), and diastolic blood pressure (DBP) were recorded every 5 min until the end of the procedure. The time required to achieve T4 level was recorded. The motor block in the lower limbs was assessed by the modified Bromage scale: 0, free movement of legs and feet; 1, just able to flex knees with free movement of feet; 2, unable to flex knees but with free movement of feet; and 3, unable to move legs or feet. For patients in which a satisfactory block level was not achieved by the spinal injection after 15 min or if systemic analgesics did not control any intraoperative pain, patients were given general anesthesia (GA) and were excluded from the study. Patients were advised about the possibility to convert to GA if they were dissatisfied with the block they received. Every patient received Inj. Midazolam 1 mg with Inj. Butorphanol 0.5 mg for sedation. The overall quality of intraoperative muscle relaxation as poor, fair, good, and excellent was evaluated by the surgeon at end of the procedure. Patient satisfaction was evaluated after discharge from Post Anesthesia Care Unit (PACU) and classified as very satisfied, average satisfaction, or dissatisfied [5]. 

Hypotension was defined as SBP≤ 90 mmHg or ≥ 20% decrease from baseline values. It was treated by I.V. fluid bolus and vasopressors, i.e., Inj. Phenylephrine 10 µg bolus. Bradycardia was defined as HR≤ 60 beats/min and was treated by a bolus of Inj. Atropine 0.6 mg intravenously. Intra and post-operative complications were also recorded, e.g., nausea vomiting, pain, headache, etc.

In the postanesthetic care unit (PACU), the sensory level of the block was assessed every 15 min, and the time until complete regression of the block was recorded. The degree of motor block was assessed at the same time points. Patients were discharged from PACU after total regression of block, provided that postoperative pain was well controlled by systemic analgesics. Patient satisfaction was evaluated after discharge from PACU and classified as totally satisfied, average satisfaction, or not satisfied. The incidence of any complications in a postoperative period like post-dural puncture headache and postoperative urine retention was also observed and recorded. Discharge from the hospital was after patients passed urine and when cleared by the surgeon as complication free.

## Results

**Figure 1:**
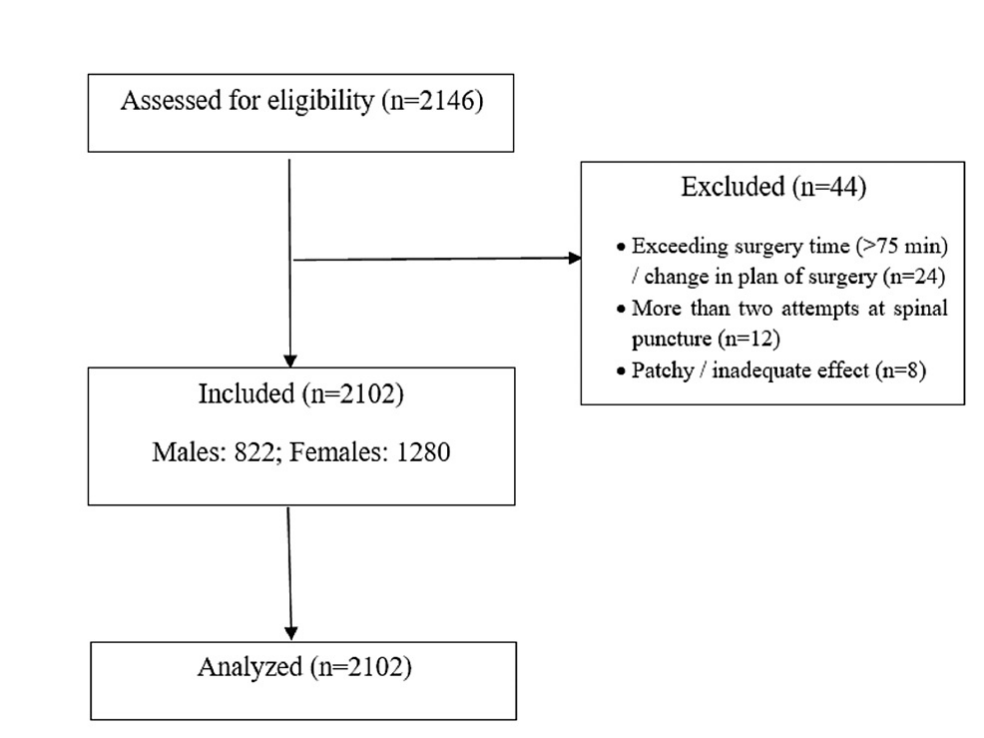
STROBE flow diagram

Among these patients, 44 patients dropped out of the study due to pre-defined criteria (Figure 1). Out of 2,102 patients included in the study, 1,280 (60.9%) were female and 822 (39.1%) were males (Figure 1). Table 1 depicts the reason behind the dropping out of the 44 patients. Most of the patients (24) were dropped due to either the exceeding surgery time (>75 min) or a change in the plan of surgery (Figure 1).

Table 1 depicts the demographic profile of the patients in the study. The mean age of the patients was 49.65 ± 6.58 years. The mean weight and height of the patients were 65.87±8.52 kg and 162.63±7.63 m, respectively. However, the body mass index was 24.82±1.81 kg/m^2 ^(Table 1).

**Table 1:** Demographic characteristics BMI: Body Mass Index; ASA: American society of Anaesthesiologists

Characteristics	Minimum-Maximum	Data as Mean± S.D
Age (in years)	40-66	49.65 ± 6.58
Weight (in kilograms)	52-84	65.87±8.52
Height (in meters)	150-180	162.63±7.63
BMI (in kg/m^2^)	21.36-28.65	24.82±1.81
ASA Grading (I:II)	1353 (64.36%):749 (35.64%)	
Comorbidity	749 (35.64%)	

All the blocks were performed at T10-T11 intervertebral space by median approach and the block was successful in all the patients. The frequency of intraoperative adverse events was also recorded. There was a single episode of hypotension in 378 patients (18.0%) and bradycardia in 273 (13.0%) patients (Table 2). All these patients responded well to a single dose of vasopressor (Ephedrine 5 mg) and Inj. Atropine (0.6mg) and there was no repeated incidence of hypotension or bradycardia in these patients. Some of the patients complained of intraoperative nausea (10.0%) and vomiting (2.5%) which responded satisfactorily to a single dose of antiemetic. There was a minimal incidence (6.0%) of complaints of shoulder tip pain for which the patients were counseled (Table 2). The majority of patients who reported average satisfaction were among this group. We also observed an unusual occurrence (5.0%) of nasal congestion in a small subset of patients, an observation not seen in other studies on segmental spinal anaesthesia at the midthoracic level (Table 2). There was no incidence of any nausea, vomiting, headache and urinary retention in the postoperative period. The block was performed in a single attempt in the majority of patients (92.0%), with a few (8.0%) requiring a second attempt. The incidence of paresthesia during needle insertion (5.8%) was recorded, whereas no patient complained of paresthesia during drug injection (Table 2).

**Table 2:** Intraoperative adverse effects

Adverse effects/Intraoperative	Frequency	Percentage
Hypotension	378	18.0
Bradycardia	273	13.0
Nausea	210	10.0
Vomiting	53	2.5
Shoulder tip pain	126	6.0
Nasal Congestion	105	5.0
Paresthesia during needle insertion	122	5.8
Paresthesia during drug injection	0	0.0

The majority of patients (94.0%) were very satisfied with the procedure and were comfortable during surgery with a quick postoperative recovery (Table 3). Some of the patients reported average satisfaction, among which most of the patients were those who experienced episodes of shoulder tip pain and nausea. None of the patients reported any post-dural puncture headache or any difficulty in resuming activity in the post-operative period (Table 3).

**Table 3:** Patient satisfaction

Patient Satisfaction	Frequency	Percentage
Very Satisfied	1,976	94.0
Average Satisfaction	126	6.0
Dissatisfied	0	0.0
Total	2,102	100

Table 4 depicts the outcome characteristics of the patients. The time for full regression of motor and sensory block range from 250-295 and 125-150 minutes. Most of the patients (1939) were discharged on the second day. The median postoperative stay time in the hospital of the patients was 31 days; however, 1,944 patients were ambulatory on the same day (Table 4).

**Table 4:** Outcome characteristics (n): number

Time to full block regression (in minutes)	267 (250-295)
Time for regression of sensory block (in minutes)	136 (125-150)
Discharge (same day:day 1:day 2) (n)	163:1939:0
Postoperative time in hospital (in hours) (n)	31 (22–38)
Ambulation: Day 0:1	1944:158

## Discussion

Laparoscopic cholecystectomy has become the standard treatment of choice in patients with symptomatic cholelithiasis. Laparoscopic approach, though advantageous poses unique challenges for an anesthesiologist perioperatively. GA was widely used for laparoscopic surgeries earlier, but LSA also gained wide popularity during the last two decades. Putensen et al. [6] in his research stated that the return of pulmonary functions to normal levels may take around 24 hours after surgery is performed by laparoscopic approach under GA. The basic mechanism in play after regional anesthesia is by blockade of afferent neural pathways along with various humoral mediator cascade systems, which manifests as a reduction in surgical stress by providing extensive pain relief. Spinal anesthesia is considered more advantageous over GA for many factors which include no airway manipulation, profound muscle relaxation, reduced incidence of DVT, good postoperative analgesia, and preservation of consciousness.

Segmental TSA was considered to be a controversial technique for a long period, owing to the fear of anesthesiologists of potential spinal cord damage and hemodynamic instability due to blockade of thoracic cardioacceleratory fibers (T2-T6) and weakness of thoracic and abdominal muscles helping in respiration. Kiran and Sweta reported a case study of a patient with Byssinosis undergoing nephrectomy under TSA and stated that the concern with TSA affecting ventilation adversely due to higher levels of the block is minimal because the use of a small dose of drug injected preserves the coughing ability of the patient and the diaphragm function remains intact as it is innervated from the cervical level (C3, 4,5) [7]. In our observation also there was no evidence of any respiratory embarrassment in any of the patients.

The advent of a new era in TSA started when a case report was published by Van Zundert et al. where a successful Laparoscopic Cholecystectomy was performed by utilizing thoracic CSE technique, with a dose of local anesthetic without any adverse event [8]. This patient was a chronic smoker, with severely compromised respiratory function (COPD with severe emphysema with homozygote α-1-antitrypsin deficiency). He was dependent on oxygen support and had very low functional capacity (dyspnea even on mild exertion). Van Zundert et al. also conducted a study on 20 patients, using a very similar anesthesia technique, and suggested that segmental spinal anesthesia can be used for laparoscopic surgeries in other healthy patients [9]. Our study also substantiated the fact that segmental thoracic spinal is a safe alternative to traditionally followed forms of anesthesia, with various advantages and minimal or no complications.

The most dreaded complication feared by anesthesiologists while performing a thoracic puncture is the potential of damaging the spinal cord. The results of MRI studies indicate that in the thoracic segment, the position of the spinal cord is anterior in the thecal sac; thus, a safe distance is present at the thoracic level preventing spinal needle contact with the neural tissues. An anatomical clarification was formulated by Imbelloni and Gouveia after studying MR of the thoracic spinal canal in 50 patients and explaining the absence of spinal cord injury even during accidental perforation of the thoracic dura mater [10]. They concluded that the space between the dura mater and spinal cord in the thoracic region measured with MR imaging was 5.19 mm at T2, 7.75 mm at T5, and 5.88 mm at T10. MR imaging confirmed that the cord and the cauda equine lie in close proximity to the dura mater posteriorly in the lumbar region, but they lie anteriorly in the thoracic region of the spinal cord. These anatomical landmarks suggested that there is a safe distance that allows the advancement of a needle to a point without touching the cord, even during accidental perforation of the dura mater while performing spinal anesthesia. They also stated that the incidence of paresthesia during lumbar spinal needle insertion attempts is around 13.6%. In our study, we recorded an incidence of paresthesia of 5.8%, which is lower than their reported incidence, but there was no post-operative sequelae or complication.

Hogan et al. studied 12 autopsy adult subjects determining that thoracic nerve roots are thin leading to the effective blockade, whereas low lumbar and sacral roots are larger and resistant to blockade [11]. The amount of CSF is more in lumbar and cervical levels as compared to a thoracic level resulting in less drug dilution and dense block [12]. In all our cases that underwent a successful procedure, we observed that the effect of regional anesthesia was dense with good abdominal muscle relaxation reported by the surgeon.

Yousef et al. in their study on 90 patients undergoing Laparoscopic cholecystectomy divided into equal groups, compared GA, TSA, and LSA for the procedure [13]. The authors used (hyperbaric bupivacaine 15 mg, and fentanyl 25 mcg at L2/L3) for the Lumbar level group, and (hyperbaric bupivacaine 7.5 mg, and fentanyl 25 mcg at T10/T11) for the Thoracic level group. Tracheal intubation facilitated by standard medications was used for the GA group. Intraoperative vital parameters, any additional analgesic requirement, postoperative recovery period adverse effects, and the good satisfaction level of both surgeon and patient were evaluated. They strengthened the fact that both the regional anesthesia approaches, i.e., lumbar and thoracic are safe alternatives to GA for laparoscopic cholecystectomy in otherwise healthy patients. The postoperative analgesia was better in regional anesthesia groups as compared to the GA group. Segmental TSA as compared to lumbar spinal maintains better hemodynamic stability with the decreased requirement of vasopressors with the possibility of early ambulation and discharge. They also stated that the degree of patient satisfaction was more in TSA, thus making it an excellent choice for daycare surgery.

Paliwal et al. in a study compared segmental spinal versus GA for laparoscopic cholecystectomy on 60 patients and concluded that segmental spinal can prove to be a better choice in patients particularly with respiratory co-morbidities, with lower incidence of postoperative pneumonia and atelectasis [14]. Ellakany et al. conducted a randomized controlled study in 60 patients planned for open surgeries due to abdominal malignancies comparing segmental thoracic spinal and GA [15]. They concluded that thoracic spinal is an adequate option for such high-risk patients with shortened recovery time, higher patient satisfaction, lower incidence of nausea and vomiting, and shortened hospital stay. In the present study, 94% patients were very satisfied after the surgery with the anesthesia technique. 

In the present study, one of the limitations is that sadly our satisfaction score is not in such a way as by Goth et al. [16]. They suggested that the assessment of patient satisfaction implies multiple aspects. The study would be more impactful if the different aspects of patient satisfaction would have been taken care of. Moreover, the high incidence of hypotension and bradycardia and their impact on vital organs need further evaluation.

## Conclusions

TSA can be considered a practically feasible alternative to conventional forms of anesthesia for patients planning to undergo Laparoscopic Cholecystectomy. It has the advantage of providing minimal postoperative complications, and a higher degree of patient satisfaction. It also provides good sensory and motor block with no evidence of postoperative neurological complications. However, the anesthetist needs to be careful during hemodynamic monitoring.
